# Pro-inflammatory Cytokines Drive Deregulation of Potassium Channel Expression in Primary Synovial Fibroblasts

**DOI:** 10.3389/fphys.2020.00226

**Published:** 2020-03-24

**Authors:** Omar Haidar, Nathanael O’Neill, Caroline A. Staunton, Selvan Bavan, Fiona O’Brien, Sarah Zouggari, Umar Sharif, Ali Mobasheri, Kosuke Kumagai, Richard Barrett-Jolley

**Affiliations:** ^1^Institute of Ageing and Chronic Disease, University of Liverpool, Liverpool, United Kingdom; ^2^Research Unit of Medical Imaging, Physics and Technology, University of Oulu, Oulu, Finland; ^3^Department of Regenerative Medicine, State Research Institute Centre for Innovative Medicine, Vilnius, Lithuania; ^4^Department of Orthopedics and Department of Rheumatology & Clinical Immunology, UMC Utrecht, Utrecht, Netherlands; ^5^Versus Arthritis Centre for Sport, Exercise and Osteoarthritis Research, Queen’s Medical Centre, Nottingham, United Kingdom; ^6^Department of Orthopaedic Surgery, Shiga University of Medical Science, Shiga, Japan

**Keywords:** TNFα, IL1β, inflammation, synovial fibroblast, ion channel

## Abstract

The synovium secretes synovial fluid, but is also richly innervated with nociceptors and acts as a gateway between avascular joint tissues and the circulatory system. Resident fibroblast-like synoviocytes’ (FLS) calcium-activated potassium channels (*K*_Ca_) change in activity in arthritis models and this correlates with FLS *activation*.

**Objective:**

To investigate this activation in an *in vitro* model of inflammatory arthritis; 72 h treatment with cytokines TNFα and IL1β.

**Methods:**

FLS cells were isolated from rat synovial membranes. We analyzed global changes in FLS mRNA by RNA-sequencing, then focused on FLS ion channel genes and the corresponding FLS electrophysiological phenotype and finally modeling data with ingenuity pathway analysis (IPA) and MATLAB.

**Results:**

IPA showed significant activation of inflammatory, osteoarthritic and calcium signaling canonical pathways by cytokines, and we identified ∼200 channel gene transcripts. The large *K*_Ca_ (BK) channel consists of the pore forming Kcnma1 together with β-subunits. Following cytokine treatment, a significant increase in Kcnma1 RNA abundance was detected by qPCR and changes in several ion channels were detected by RNA-sequencing, including a loss of BK channel β-subunit expression Kcnmb1/2 and an increase in Kcnmb3. In electrophysiological experiments, there was a decrease in over-all current density at 20 mV without change in chord conductance at this potential.

**Conclusion:**

TNFα and IL1β treatment of FLS *in vitro* recapitulated several common features of inflammatory arthritis at the transcriptomic level, including increase in Kcnma1 and Kcnmb3 gene expression.

## Introduction

Rheumatoid arthritis (RA) and osteoarthritis (OA) are degenerative diseases that target articular joint structures resulting in pain, loss of function and frequent disability. Whilst RA is an established inflammatory condition, the contribution of inflammatory processes to OA was less well known until recently. Mediators of inflammation (i.e., including pro-inflammatory cytokines) contribute to the development of synovitis, which is known to drive disease progression in RA. In contrast, it is thought that in OA, synovitis can be caused by the release of cartilage fragments and meniscal damage that in turn, activate synovial lining cells (Fernandez-Madrid et al., 1995; Roemer et al., 2013; Mathiessen and Conaghan, 2017). How inflammation drives joint destruction is not fully known. One feature of synovitis, however, is the presence of major pro-inflammatory cytokines, such as interleukin-1β (IL-1β), tumor necrosis factor alpha (TNFα), and interleukin 6 (IL-6), resulting in suppression of collagen and proteoglycan synthesis, increased inflammatory signaling, and protease expression and activation (Martel-Pelletier et al., 1999).

Management of arthritis has significantly improved in recent years’, however, remission is rarely achieved, and many patients remain unresponsive to conventional and/or biologic treatments. In addition, current therapies and treatments are associated with notable side effects that can pose great challenges for long-term treatment, especially in patients with cardiovascular co-morbidities. For example, some drugs can increase the risk of cardiovascular disease or significantly impair immune responses, rendering patients more susceptible to infections and cancer (Kahlenberg and Fox, 2011). Therefore, new therapeutic options and novel targets are needed that lead to pronounced improvement without inducing unwanted side effects and thus avoiding the need for joint replacement.

The synovium is the major barrier between the joint and the systemic circulation and plays a role in maintaining the health of articular cartilage (Sutton et al., 2009; Berenbaum, 2013). The synovium lubricates the articular surfaces and provides nutrients for chondrocytes within the avascular cartilage; it has been suggested that catabolic enzymes such as matrix metalloproteinase (MMPs) are produced by synovial cells and diffuse into the cartilage. The intimal lining layer of the synovium produces lubricious synovial fluid and is composed of two cell types in relatively equal proportions: Type A or macrophage-like synovial cells and Type B or fibroblasts like synoviocytes (FLS). FLS cells contribute to the structural integrity of the joints by controlling the composition of the synovial fluid and extracellular matrix (ECM) of the joint. The synovial environment changes physically, chemically, and physiologically with injury or the onset of disease and is thought to be a mediator in arthritis pain (Grubb, 2004; Sellam and Berenbaum, 2010; Kumahashi et al., 2011). FLS cells have been implicated in arthritis as they exhibit a transformed phenotype with increased invasiveness and production of various pro-inflammatory mediators that perpetrate inflammation and proteases that contribute to cartilage destruction (Noss and Brenner, 2008; Bartok and Firestein, 2010). Understanding the biology and regulation of FLS cells provides insight into the pathogenesis of inflammatory arthritis. FLS cells could potentially be targeted pharmacologically to produce increased volumes of synovial fluid as an alternative to intra-articular hyaluronan or synthetic fluid injection therapies. They are also a plausible analgesic target because they may interact with sensory neurons and have been described as “amplifiers” of neuropeptide mediated inflammation and pain.

To deepen our understanding of the synovium in the context of synovial joint health and disease, the electrophysiological profile of FLS cells needs to be characterized, along with the ion channels that are present. Ion channels are an essential component of any cell membrane that controls ion movement in and out of the cell and play an important role in a multitude of cell regulating processes, typically by modulating the membrane potential. Electrophysiological techniques have been used to characterize the biophysical properties of a number of different FLS preparations, including mouse, rabbit, bovine and human (Large et al., 2010; Friebel et al., 2014; Clark et al., 2017). The best available whole-cell mathematical model of the FLS is heavily dominated by a Ca^2+^-activated potassium conductance, with small added components of inward rectifiers, background and leak. A recent study by Kondo et al. (2018) demonstrated that human FLS express high levels of Ca^2+^-activated potassium channels and these ion channels were also identified in both RA-derived and rodent model FLS studies. Typically, Ca^2+^-activated channels couple with Ca^2+^ entry channels such as transient receptor potential (TRP) channels; they are both activated by the Ca^2+^ ions that enter and maintain the membrane potential hyperpolarized to “draw in” further Ca^2+^. In FLS, Ca^2+^-activated potassium channels appear to drive invasiveness of synoviocytes and progression of arthritis in both human and rodent RA models (Petho et al., 2016; Tanner et al., 2019), by increasing production of both inflammatory mediators and catabolic enzymes (Hu et al., 2012; Friebel et al., 2014; Tanner et al., 2015). This is a paradoxical effect for a potassium conductance, that would be predicted to hyperpolarize cells and reduce migration, proliferation and activity in general.

The synovium is an obvious target for the development of novel interventions in both RA and OA. The role of synovitis, the low-grade inflammation of the synovial lining of the joint, in OA progression is gradually emerging. Therefore, in this work we investigate the pathophysiology of cytokine induced synovitis in cultured synovial cells.

We investigate whether the TNFα and IL1β cytokine *in vitro* model of inflammation leads to a significant change of the BK ion channel and quantify the mechanism of this change. We use a combination of next generation RNA sequencing (NGS), qPCR, and patch-clamp electrophysiology to uncover several changes in potassium channel gene expression together with changes in cellular phenotype which involves a phenotypic switch in response to inflammation.

## Materials and Methods

Further methodological details are included in the Supplementary Methods section (see Supplementary File S2).

### Animals

Synovial cells were prepared from tissue from rats euthanized by Home Office Approved methods for unassociated reasons in line with the ARRIVE Guidelines. All rats were untreated/wild-type male adult Wistar.

### Preparation of FLS Cells

Synovial fibroblasts were isolated from rat knee joints as described previously. Briefly, patella and menisci with attached synovial membranes were isolated and placed in 12-well plates in low glucose DMEM (Thermo Fischer, United Kingdom) with 20% fetal bovine serum (FBS), 100 U/ml penicillin, 100 μg/ml streptomycin and 2.5 μg/ml amphotericin B (Thermo Fischer, United Kingdom) at 37°C in a 5% CO_2_ incubator. For the first 7 days medium was replaced daily whilst out-growing FLS emerged from the tissues. At 7 days residual tissue was discarded and cells cultured as normal; when confluent, cells were detached from the flask surface by 1% Trypsin-EDTA solution. The suspension was centrifuged (340 × *g*, 5 min) and the resulting pellet was resuspended in culture medium (as above).

### Immunohistochemistry

In brief, cell suspension at a density of 2.5 × 10^4^ cell/ml was transferred to multiwall plates and fixed with 2% paraformaldehyde in PBS at room temperature. CD248 (an FLS marker) immunohistochemistry was performed using rabbit anti-CD248 primary antibody (Ab, 1:100 dilution; Abcam, Cambridge) and FITC-conjugated donkey Anti-rabbit IgG secondary antibody (Ab, 1:500 dilution; Jackson ImmunoResearch Laboratories). Non-specific binding was blocked as previously described (Mobasheri et al., 2005). After 24 h at 4°C, cells were washed three times for 5 min with 0.05% SSC-20 and 0.005% Triton-X100. Slides were finally dipped into distilled water, air dried, and mounted with mounting media (Vectashield with DAPI). Cells were visualized with confocal microscopy.

### Real−Time PCR

RNA extraction was carried out using the RNeasy Plus Micro kit, together with gDNA eliminator and MinElute spin columns (Qiagen, United Kingdom). cDNA synthesis (mRNA) was performed using the RT2 First Strand kit (Qiagen, Netherlands) according to the manufacturer’s protocol. qPCR analysis was performed using the Stratagene MX3000P RT-PCR System (Stratagene, La Jolla, CA, United States) in a 25−μL reaction mixture. Expression relative to housekeeper Rplp1 was calculated as ΔCt.

### RNA “Next Generation Sequencing” (NGS)

**FIGURE 1:**
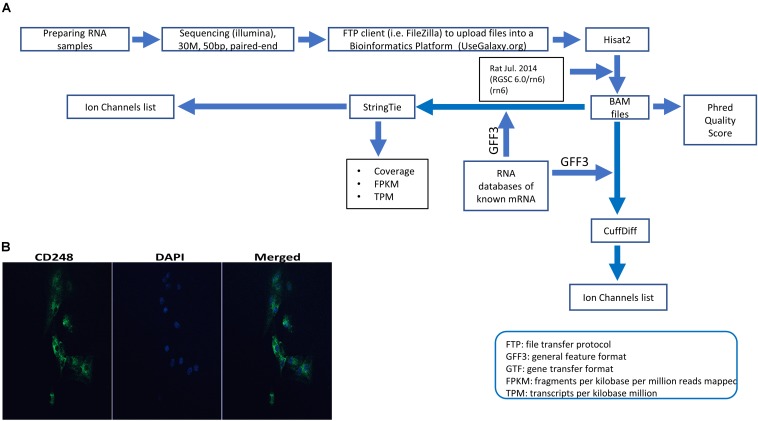
Experimental pipeline and verification. **(A)** Schematic of the next generation sequencing (NGS) pipeline. RNA was extracted from synovial tissue from eight animals, were split into control and test groups and the test samples were treated with 10 ng IL1β and TNFα as described in the section “Materials and Methods.” These eight samples sequenced with Illumina and raw data files were uploaded using a FTP client to a bioinformatics platform for analysis. Within the bioinformatics platform, reads were aligned/mapped with Hisat2 program using the reference genome Rat Jul. 2014 (RGSC 6.0/rn6) (rn6). The resulting BAM files were used to measure phred quality score for all samples. Also, BAM files along with an annotation file (Rat GFF3) to assemble mapped reads and quantify gene expression. Such assembly and quantification took place with StringTie and CuffDiff package/tool that generated gene abundance estimates and differentially expressed genes, respectively. After that, using several servers and a local custom-script in MATLAB software allowed us to filter the transcriptome for all known ion channel genes for further analysis (for example, see Tables 1, 2). **(B)** Expression of CD248 in FLS cells. Immunofluorescence FLS cells showing the FLS marker CD248. DAPI was used for nuclear counterstaining (see Supplementary Methods in Supplementary File S2 for details).

Figure 1 summarizes our NGS workflow. In brief, cells were treated with 10 ng/ml of both TNF-α + IL1β (TNF-α from Thermo Fischer, United Kingdom and IL1β from R&D systems) for 72 h. RNA extraction was carried out using the RNeasy Plus Micro kit (Qiagen, United Kingdom) according to the manufacturers protocol. Any RNA samples with concentrations of less than 5 μg/ml and/or purity (260/280 and 260/230) less than 1.8 were excluded. RNA samples were sent to GATC-Biotech, Germany for sequencing. Samples were read as paired-end with a sequencing depth of 30 M and a read length of 50 bp. Further details are included in Supplementary Methods (see Supplementary File S2). Further bioinformatics were performed with R, for example the DAPCA package or our local installation of the Galaxy server suite (Afgan et al., 2018). Specific packages are mentioned in the text, Supplementary Methods (see Supplementary File S2) and Figure 1. *Full data are available on the EBI array express database with accession number: E-MTAB-7798*.

**TABLE 1:** FLS channel gene RNA expression lower after cytokine (10 ng/ml TNFα and IL1β) treatment.

Gene	Official name	Log_2_ (95% CI)
Kcnj15	Potassium inward rectified channel subfamily j member 15	−5.51 (−9.57 to −2.48)
Ano2	Anoctamin 2	−2.59 (−5.5 to 0)
Kcnb1	Potassium voltage-gated channel subfamily b member 1	−2.44 (−1.85 to −1)
Kcnv2	Potassium voltage-gated channel modifier subfamily v member 2	−2.4 (−4.85 to 0)
Kcnip1	Potassium voltage-gated channel interacting protein 1	−2.36 (−3.88 to −0.88)
Kcnmb1	Potassium calcium-activated channel subfamily m regulatory beta subunit 1	−1.98 (−3.28 to 0.69)
Kcns1	Potassium voltage-gated channel modifier subfamily s member 1	−1.88 (−6.84 to −0.42)
Kcnj8	Potassium inward rectifier j member 8 (Kir6.1)	−1.79 (−4.71 to 2.17)
Kcnd3	Potassium voltage-gated channel subfamily d member 3	−1.78 (−2.63 to 0.29)
Nalcn	Sodium leak channel, non-selective	−1.77 (−3.43 to −0.85)
Clca2	Chloride channel accessory 2	−1.76 (−6.72 to 0.65)
Kcne3	Potassium voltage-gated channel subfamily e regulatory subunit 3	−1.67 (−1.84 to −0.15)
Asic1	Acid sensing ion channel subunit 1	−1.64 (−6.44 to 0)
Clcn4	Chloride voltage-gated channel 4	−1.61 (−2.61 to −0.95)
Trpm8	Trpm8 channel associated factor 1	−1.61 (−2.09 to −0.99)

*Qualification cytokine log_2_ (geometric mean) < −1.5 of control (= approximately 33%), *n* = 4 control and 4 cytokine treated samples (from four animals).*

**TABLE 2:** FLS channel gene RNA expression increased after cytokine (10 ng/ml TNFα and IL1β) treatment.

Gene ID	Official name	Log_2_ (95% CI)
Kcnc1	Potassium voltage-gated channel subfamily c member 1	4.32 (0.68 to 5.93)
Kcnc3	Potassium voltage-gated channel subfamily c member 3	3.46 (0.76 to 5.94)
Trpc3	Transient receptor potential cation channel subfamily c member 3	3.06 (3.73 to 6.5)
Catsper3	Cation channel sperm associated 3	2.83 (0.14 to 5.75)
Kcng3	Potassium voltage-gated channel modifier subfamily g member 3	2.81 (2.77 to 6.65)
Tmc7	Transmembrane channel like 7	2.77 (1.99 to 5.53)
Gjc3	Gap junction protein gamma 3	2.75 (0.03 to 4.35)
Scnn1g	Sodium channel epithelial 1 gamma subunit	2.56 (2.77 to 5.45)
Asic2	Acid sensing ion channel subunit 2	2.52 (0.66 to 4.65)
Kcnmb3	Potassium calcium-activated channel subfamily m regulatory beta subunit 3	2.34 (0 to 4.73)
Cracr2a	Calcium release activated channel regulator 2a	2.32 (0.01 to 1.96)
Kcnn3	Potassium calcium-activated channel subfamily n member 3	2.07 (0.57 to 4.09)
Tmc4	Transmembrane channel like 4	1.94 (0.27 to 2.47)
Asic4	Acid sensing ion channel subunit family member 4	1.75 (0.52 to 2.76)
Kcnj11	Potassium inward rectifier j member 11 (Kir6.2)	1.74 (0 to 6.56)
Trpv6	Transient receptor potential cation channel subfamily v member 6	1.7 (0.01 to 5.43)
Hvcn1	Hydrogen voltage gated channel 1	1.69 (0.98 to 2.84)
Trpv1	Transient receptor potential cation channel subfamily v member 1	1.61 (0.98 to 2.07)
Kcnk7	Potassium two pore domain channel subfamily k member 7	1.6 (−1.85 to 1.03)
Cacna1d	Calcium voltage-gated channel subunit alpha1 d	1.57 (0.32 to 2.09)
Asic5	Acid sensing ion channel subunit family member 5	1.55 (0 to 5.78)

*Qualification cytokine log_2_ (geometric mean) > 1.5 of control (= approximately 300%), *n* = 4 control and 4 cytokine treated samples (from four animals).*

### Electrophysiology

Electrophysiology was performed as described previously (Lewis et al., 2013) but using isolated FLS. Intracellular solution was 115 mM gluconic acid/potassium salt, 26 mM KCl, 1 mM MgCl_2_ (BDH, VWR International Ltd.), 5 mM Ethylene glycol tetraacetic acid (EGTA), 10 mM HEPES, pH 7.2. Extracellular (bath) solution was 140 mM NaCl, 5 mM KCl, 2 mM CaCl_2_ (Fluka Analytical cat#: 21114), 1 mM MgCl_2_, 10 mM HEPES, and 5 mM Glucose, osmolality approximately 300 mOsm, pH 7.4. Junction potential −14.4 mV (Lewis et al., 2013). Thick-walled patch-pipettes were pulled from borosilicate glass capillary tubes (outer diameter 1.5 mm, inner diameter 0.86 mm; Intracel, United Kingdom) and gave a resistance of ∼8 MΩ when filled. Whole cell patch-clamp electrophysiology was performed on FLS cells using a Cairn Optopatch amplifier (Cairn Research, United Kingdom). To compare voltage-gated currents, we performed whole-cell patch clamp experiments with voltage steps starting from a holding potential of −80 mV for 2 s. Recordings were filtered at 1 kHz, digitized at 3 kHz and recorded on a computer using WinWCP 5.3.4 software (John Dempster, Strathclyde University, United Kingdom). All experiments were performed at room temperature (18–22°C), and the results are expressed as the mean ± SEM.

Analysis was performed using WinWCP 5.3.4 software (John Dempster, Strathclyde University, United Kingdom). Boltzmann curve fits were computed in MATLAB through non-linear least squares optimization.

## Results

### Enriched Pathways in FLS Cells After Cytokine Treatment

**FIGURE 2:**
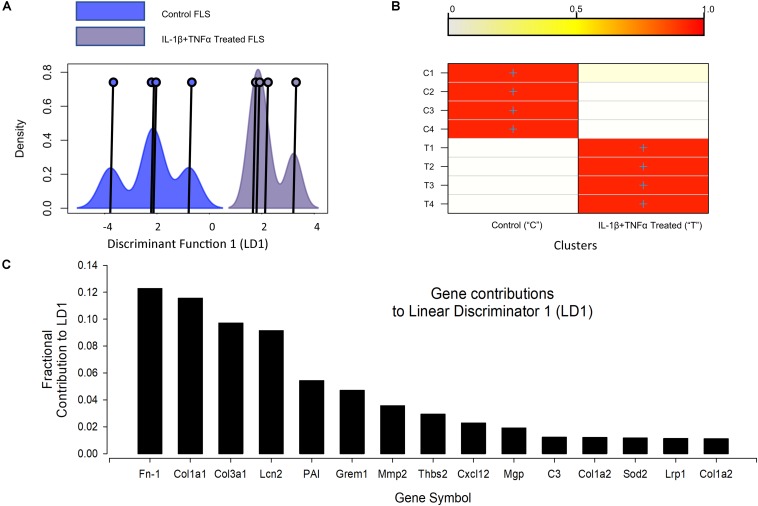
Discriminant analyses of global IL-1β TNFα treatment effects. **(A)** Shows the kernel density plots of the discriminant component co-ordinates for the control and IL-1β/TNFα treatment groups; co-ordinates on the *x*-axis and density on the *y*-axis; Individual co-ordinate center points are illustrated by the vertical line and circle. There is clear separation between control and cytokine treated FLS samples. **(B)** A graphical confusion matrix showing the actual group membership (*y*-axis) and predicted cluster membership (on the *y*-axis). All groups are correctly clustered with greater than 0.9 probability. **(C)** The top 15 contributors to the linear discriminator function: Fn-1, fibronectin 1; Col1a1, collagen type I alpha 1 chain; Col3a1, collagen type III alpha 1 chain; Lcn2, lipocalin 2; PAI, serpine1; Grem1, gremlin 1; Mmp2, matrix metallopeptidase 2; Thbs2, thrombospondin 2; Cxcl12, C-X-C motif chemokine ligand 12; Mgp, matrix Gla protein; C3, complement C3; Col1a2, collagen type I alpha 2 chain; Sod2, superoxide dismutase 2; Lrp1, LDL receptor related protein 1; Col1a2, collagen type I alpha 2.

RNA-seq detected 33251 transcripts and the *full data are available on the EBI array express database with accession number: E-MTAB-7798.* The top (highest FPKM) expressed 10 genes were similar between control and cytokine treated cells (Supplementary Tables S1, S2). Our first bioinformatic analysis tested the reproducibility of the 72 h, 10 ng/ml TNFα + IL1β treatment. We used discriminant analyses of principal components (DAPC) with in the DAPC package to show good separation of the two populations (Figure 2); the genes primarily discriminating the treatment and control populations are largely those well established to be important for joint function, including several collagens and a matrix metallopeptidase (MMP2). Ingenuity pathway (IPA) analysis (Qiagen, United Kingdom) was then used to identify the upstream regulators of the global differential expression pattern. This analysis predicted the top two regulators to be TNFα and IL1β (*p*-values 3e-17 and 6e-13, respectively), this is unsurprising since this was indeed the treatment regimen.

**FIGURE 3:**
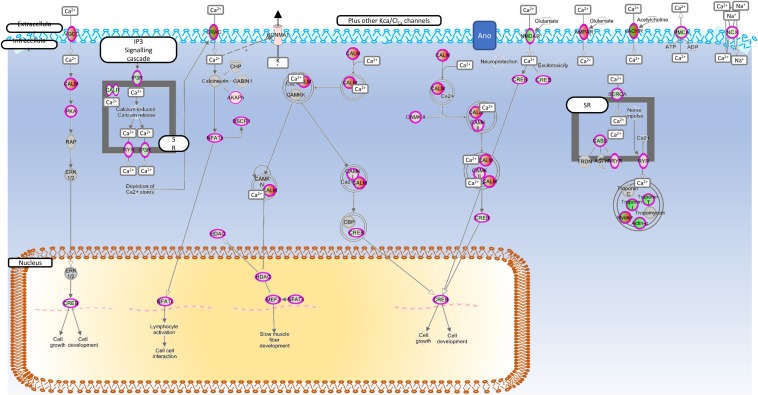
Enriched calcium regulation pathway. Genes mapped to the calcium signaling canonical pathway by IPA. Red, increased expression; green, decreased expression (for interpretation of the references to color in the figure legend, please refer to the online version of this article).

Figure 3 shows the canonical calcium signally pathway (*p* < 0.5e-7), which was enriched following cytokine treatment. In addition, the rheumatoid (*p* < 5e-13) and OA (*p* < 1e-9) pathways and cellular movement and proliferation canonical pathways (predicted *activation*, *p* < 1e-13 in both cases) were also enriched following cytokine treatment (data not shown). In all cases, TNFα was determined to be the top *causal* agent, but four ion channels were also significant causal regulators (adjusted *p* < 0.05) of the transcript-wide treatment changes; Clcn5, Trpv4, Trpv1, Kcnn4.

### Next Generation RNA Sequencing Analysis of Ion Channel Gene Expression

Initial RNA-seq experiments were intended to give a transcriptome wide, unbiased, assessment of ion channel changes in cytokine treated FLS. We identified 190 channel genes, including porins, connexins and ion-channel isoform genes (including α- to ε-subunits), but excluded interacting proteins, other regulatory proteins and the so-called potassium tetramerization domain proteins. The top 50 genes, in terms of FPKM are given for control and cytokine treated datasets in Supplementary Tables S3, S4, respectively.

### Next Generation RNA Sequencing Ion Channel Differential Expression

**FIGURE 4:**
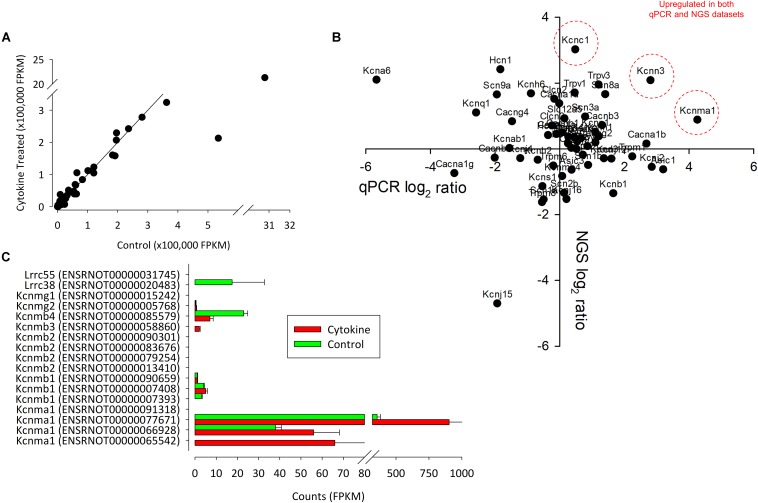
The correlation between control and NGS expression data. **(A)** We found a strong correlation between control and treated (72 h of IL1β + TNFα) channel expression data. Each point represents the intersection of four control and four test values. In **(B)**, the correlation between qPCR (ΔΔCts) and NGS ratio (FPKM values) are shown. Data suggest that there only a weak linear correlation between qPCR and NGS. *P*-value = 0.3. *R* = 0.29. *n* = 14 (eight animals for NGS and six animals for PCR). Genes that were upregulated in both datasets are indicated in red. **(C)** Summary of NGS expression of all known (rat) splice variants of Kcnma1 and associated Kcnmb subunits.

One of the key ion channels involved with regulation of FLS is the large calcium activated potassium channel (BK, Kcnmx) and it is notable that there was a reduction in expression of β-subunit Kcnmb1/2 and appearance of Kcnmb3 after cytokine treatment. We further analyzed this family transcript by transcript for the 13 known (already annotated) splice variants of these channels; Kcnma1, Kcnmb1, Kcnmb2, Kcnmb3, Kcnmb4 (Figure 4C). The most abundantly expressed of all these transcripts is Kcnma1 (ENSRNOT00000077671) with rather lower expression of any of the β-subunits and no detection of the one annotated Kcnmb4 variant. Following cytokine treatment expression of all of the detectable Kcnma1 splice variants was higher, but there was a lower abundance of Kcnmb4.

In total, 20 ion channel genes, undetectable in control conditions became detectable or ‘appeared’ after cytokine treatment (Supplementary Table S5), of which, the top expressed of these was Trpc3. Conversely, seven ion channel genes became undetectable or “disappeared” following cytokine treatment (Supplementary Table S6). Following cytokine treatment, we found an additional 15 genes to be down by −1.5 (log_2_) or more and 21 genes 1.5 (log_2_) greater than control (Tables 1, 2, respectively). We used tissue from four animals for the NGS study each animal tissue split into test and control groups; the “*n”* presented in the legends refers to the number of biological replicates (= animals).

### qPCR Verification of RNA Changes

To add further support to the unbiased RNA-seq ion channel analysis we performed qPCR on sets of control and IL1β/TNFα treated FLS with panels of Ca^2+^-potassium channels (Kcnma1, Kcnn1, Kcnn2, Kcnn3) and other ion channel genes (Figure 4B). We did not have primers for all the potassium channel genes identified by next generation sequencing. Three potassium genes were differentially expressed; two voltage-gated potassium channels Kcna6 and Kcnc2-significantly decreased (*p* < 0.05, *n* = 4,4), whereas the large calcium potassium channel Kcnma1 was upregulated (*p* < 0.05, *n* = 4,4).

### Electrophysiology

Neither RNA nor protein expression studies can confirm changes in functional ion channel expression or, functional changes resulting from post-translational changes, Furthermore, several different potassium channel isoforms were identified, of which some upregulated and some downregulated. Therefore, to investigate the effect of cytokine treatment on the electrophysiological fingerprint, we conducted functional assays of ion channel expression with patch-clamp electrophysiology. The primary changes observed in the more limited qRT-PCR data would predict over all *loss of* voltage-gated potassium ion channel activity and increase in the less voltage-dependent calcium activated potassium channels.

#### Resting Membrane Potential

Following cytokine treatment, the resting membrane potential of FLS decreased (depolarized) from −48.6 ± 1.7 to −38.6 ± 2.8 mV, junction potential corrected (data not shown, *p* ≤ 0.05, unpaired *t*-test).

#### Current Voltage Currents

**FIGURE 5:**
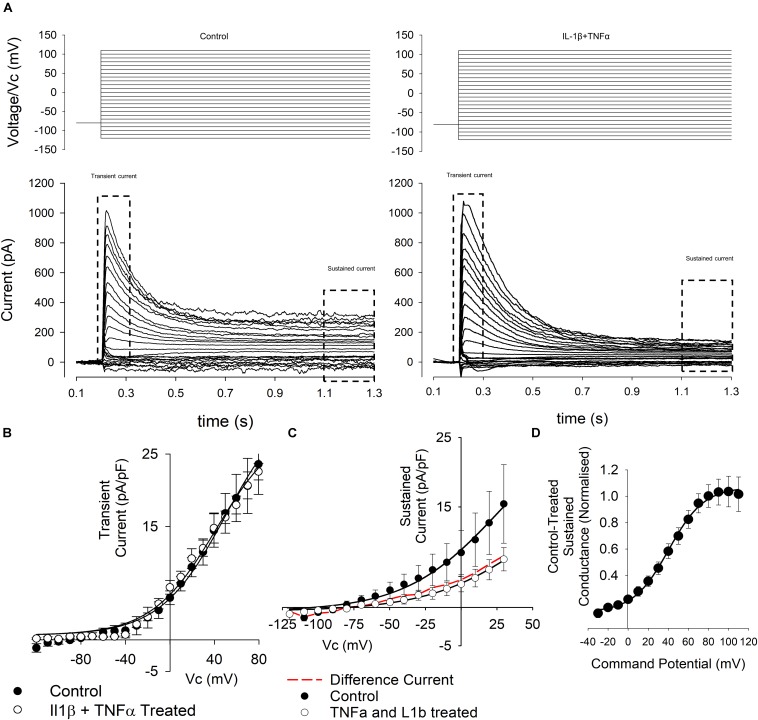
Whole-cell voltage-gated currents from control and cytokine treated FLS. **(A)** Top panels show the voltage step protocols and the evoked currents are shown below for control (left) and IL1β + TNFα (right) conditions. Note that FLS exhibit both transient and sustained currents. These phases of current were then analyzed separately as indicated. **(B)** Current–voltage curves (left) from the *transient* currents recorded in a number or experiments such as that illustrated in **(A)**. There was no significant difference between control and treated *transient* current density. Data points are shown as mean ± SEM (*n* = 18 for control and *n* = 11 for IL1β + TNFα). **(C)** Current–voltage curves from the *sustained* currents recorded in a number or experiments such as that illustrated in **(A)**. The red line in the current–voltage curve is the difference current for control-cytokine treated. **(D)** Difference conductance–voltage curve for the cytokine difference current shown in **(C)**. The line is fit with a Boltzmann, *see text*.

As seen in Figure 5A, some cells exhibited clear transient and sustained components whereas others exhibited only the sustained component of the current. We therefore analyzed the transient and sustained components of the current separately in all cases.

We found no overall change in the maximum amplitude of the transient current (Figure 5B), but the sustained current (Figure 5C) density, measured at +20 mV was decreased (12.5 ± 2.3 pA/pF to 3.8 ± 0.8 pA/pF, *n* = 24,20, *p* < 0.05). There was no change in chord conductance measured at this potential (271 ± 45 pS/pF to 120 ± 20 pS/pF, *n* = 24,20). To characterize the nature of the conductance apparently *inhibited* by cytokine treatment we calculated the difference current for cytokine treatment (i.e., cytokine-treated current; Figure 5D). This revealed a strongly voltage-gated current with mid-point for activation 40 ± 1.2 mV and slope 17.6 ± 1.2 mV, *n* = 25.

#### Pharmacological Modulation of Currents

**FIGURE 6:**
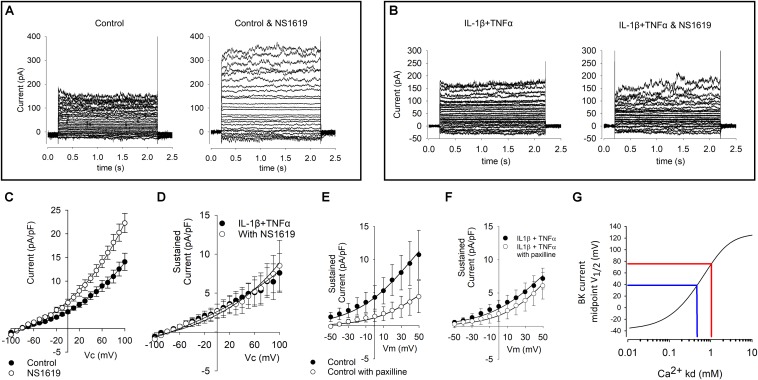
Effects of BK channel drugs on FLS whole-cell currents. **(A)** Representative examples of untreated (control) cells before (left) and in the presence of 1 μM of the BK channel opener NS1619 (left). Voltage protocols as shown in Figure 5. **(B)** Representative raw example current families of cytokine (10 ng/ml IL1β + TNFα) treated cells in the absence (left) and presence (right) of 1 μM NS1619. **(C)** Current–voltage curves from a number of control FLS cells such as that shown in **(A)**. Current is significantly greater in the presence of NS1619, *p* < 0.05, *n* = 9 and 6. **(D)** Current–voltage curves for a number of treated FLS cells such as that shown in **(B)**. These two curves are not significantly different from each other *n* = 6 and 6. **(E)** Current–voltage curves from a number of control FLS cells treated with and without paxilline; there is a significant decrease in paxilline current *p* < 0.05 *n* = 13,5), but **(F)** there was no significant different of current density in the presence of the BK inhibitor paxilline following cytokine treatment (*n* = 6, 12). **(G)** *Numerical simulation of data from Clark et al. (2017) model*. To quantify the degree of change of BK channel modulation apparently taking place with this treatment we used the model of verbatim with the exception that we varied the inherent BK channel Ca^2+^ sensitivity parameter *Kd*. In this simulation, the independent variable *Kd* is plotted on the *x*-axis and the predicted BK current midpoint (V_1__/__2_) is plotted on the *y*-axis. In blue, we have added hypothetical midpoints of 40 mV and 80 mV representing a hypothetical shift in 40 mV by cytokine treatment. The complete MATLAB code for this simulation is included in the Supplementary File S1.

Despite the *increase* of KCNMA1 RNA in both qPCR and RNA-Seq our electrophysiological data show a reduced current density. To investigate if the maximum possible BK current is altered in cytokine treatment, we repeated our standard voltage protocol (above) in the presence of 1 μM of the BK channel opener NS1619 and found a significant increase in current density in the presence of NS1619 in control cells (Figures 6A,C, *p* ≤ 0.05, *n* = 9,6), we did not see an equivalent increase in the cytokine treated cells (Figures 6B,D). We then repeated this experiment with 1 μM of the BK channel inhibitor paxilline. Untreated cell current density was significantly smaller in the presence of paxilline, but not significantly reduced in treated cells (Figures 6E,F).

## Discussion

In this study, we used an *in vitro* model of synovial cell inflammation to investigate the pathophysiology of cytokine induced synovitis. We have demonstrated that IL1β and TNFα treatment of FLS cells resulted in profound changes in arthritic, inflammatory, and Ca^2+^ regulatory pathways similar to that reported in RA models. We found differential expression of several ion channels with transcriptomics and our electrophysiological experiments show a reduction of whole-cell current density.

### The Pathophysiological Validity of the 72 h IL1β and TNFα Model

Treatment of joint tissue with TNFα and IL1β cytokines is an established *in vitro* model of inflammatory arthritis with tissue typically exposed to between 10 ng/ml of TNFα and IL1β for between 2 and 7 days. In the present study, we use the lower end of the concentration range, 10 ng and treat for 72 h (De Ceuninck et al., 2004; Stevens et al., 2008, 2009; Pretzel et al., 2009; Williams et al., 2011; Williams, 2014). This regime is hypothesized to activate inflammatory pathways, but there will be no cytokine remaining by the time of the electrophysiological experiments that could cause confounding direct effects. We treated FLS cells with the pro-inflammatory cytokines IL1β and TNFα in order to understand the cellular changes that occur when these FLS are subjected to higher-than-normal levels of these cytokines *in vivo*, for example, in arthritis. This model has distinct advantages of 3Rs, consistency, reproducibility and allowing the investigation of distinct pathways in isolation but since it is an acute model it may lack some chronic features of *in vivo* models. Our transcriptome analysis demonstrated that pathways were activated in common with arthritis movement of cells, proliferation and both RA and OA. In an *in vitro* model of RA, Tanner et al. (2019) also showed up regulation of Kcnma1 (message and protein) analogous to that observed with our 72 h cytokine treatment. Furthermore, more recent data suggests a correlation between FLS invasiveness and expression of the β-subunit (KCNMB3) in human samples of FLS from RA patients (Petho et al., 2016). *Taken together with the changes in Ca^2+^ signaling, we show that our in vitro model captures several features of the inflammatory joint phenotype and that FLS have been “activated” as observed in animal models of arthritis.*

Causal analysis is a relatively new mathematical technique that allows one to move from probability of agreement or simple correlation toward probability of causation in networks. The IPA implementation of this identified both IL1β and TNFα as master-regulators of the changes we observe and considering our experimental design included time matched controls, this strongly supports a suitable dosage and incubation time. Whilst both IL1β and TNFα were identified by IPA as “master regulators,” TNFα transcriptome-wide causation was stronger than that of IL1β. Interestingly, FLS cells harvested from RA patients exhibit a marked transient elevation of intracellular Ca^2+^ on exposure to TNFα (Yoo et al., 2006) raising the possibility that this could be the initial trigger for the resulting pathway changes. However, it should be noted that such a transient lasts less than a minute and in our study cells were challenged with cytokines 72 h prior to experiments. Furthermore, cells were replaced in cytokine-free medium for electrophysiology, so there would be no cytokine physically present at that time. Also, the previously reported TNFα induced Ca^2+^ signal was only clear in FLS from RA; it was largely absent in FLS from OA patients and not investigated in FLS from healthy controls (Yoo et al., 2006).

### Previous Studies of Differential Expression of Membrane Ion Channels in Synovium

Until very recently, little was known of the FLS ion channel compliment (the “channelome”) compared to that of the another central joint cell, the chondrocyte (Barrett-Jolley et al., 2010). One of the best-studied families of ion channels in FLS, however, is the Ca^2+^-activated potassium channel family. The high conductance member of this family, termed BK (*K*_Ca_1.1 or KCNMA1) and the “intermediate” conductance member (“IK,” or *K*_Ca_3.1) are both expressed and have roles in invasive migration, proliferation, cytokine, and MMP release (Hu et al., 2012; Friebel et al., 2014). Interestingly, inhibitors of BK decrease the signs of joint degeneration in the pristane-induced arthritis model. These channels are therefore potential drug targets to protect against joint degeneration as well as being putative biomarkers. Whilst the BK channel β- subunit (KCNMB1) was slightly increased in transcript abundance in the Lambert et al., 2014 data (similar seen by Huber et al., 2008), both of the two BK α-subunit (KNMA1) probes on the chip exhibit small decreases in expression. It should be noted that the expression of BK channel β- subunits confers modulation of ion channel activity, in many cases decreasing its sensitivity to, for example, Ca^2+^ ions (Lippiat et al., 2003; Mobasheri et al., 2012).

A recent study by Kondo et al. (2018) demonstrated that human FLS express high levels of both intermediate (*K*_Ca_3.1) and large (BK/*K*_Ca_1.1/KCNMA1) Ca^2+^-activated potassium channels. The other most highly expressed ion channels identified by Kondo et al. (2018) were KCNK2, ANO6, ANO10, and KCNK6. KCNK2/6 are members of the two-pore-domain potassium channel family and are particularly thought of as molecular sensors, whereas the ANO (anoctamin) channels are members of the large chloride channel family. This family is relatively understudied compared to potassium channels, but ANO6 (TMEM16F) is, interestingly, thought to be a Ca^2+^-activated chloride channel as well as a lipid “scramblase” (Scudieri et al., 2015), therefore, likely to be activated in parallel to Ca^2+^-activated potassium channels.

### Changes in Ion Channel Currents in the Present Study

The phenotype of FLS current recorded by whole-cell patch clamp was quite variable in terms of the presence of transient and sustained components of current, as seen in Figure 5. We found no significant changes in the transient phase of current and therefore, we focused on the sustained component of voltage activated currents that would be expected to include BK activity if it was present. Although we found a significant increase in RNA expression of the BK α-subunit gene, Kcnma1, following cytokine treatment (by qPCR), there was a decrease in over-all current density of the sustained current. To characterize the voltage-gated characteristics of the current *lost* after cytokine treatment we subtracted IV curves following cytokine treatment from the control IV curves and transformed this to a conductance-voltage curve. Since this curve fully saturated, we were able to fit it with a Boltzmann curve and derive the midpoint for activation; +40 mV. This is rather positive to most voltage-gated potassium channels, the most “positive” of which (Kcnbx/Kcncx) have activation mid-points in the +10 to +20 mV range (Coetzee et al., 1999). This cytokine sensitive conductance could be an ensemble average of a number of different ion channel changes, including increase of some and decrease of others. It could also be a BK current under conditions of low intracellular Ca^2+^ (Cui et al., 1997, 2009; Coetzee et al., 1999; Contreras et al., 2012), but, since the true local concentration of intracellular Ca^2+^ is unknown, this is difficult to assess. V_1__/__2_ for the BK channel in the virtual absence of Ca^2+^ can be much higher, for example 200–300 mV (Bao and Cox, 2005; Orio and Latorre, 2005; Wang and Brenner, 2006). The value of this parameter also depends on the nature of the co-expressed β-subunit (Coetzee et al., 1999; Contreras et al., 2012). V_1__/__2_ is typically shifted to the left in the presence of beta subunits (Brenner, 2014). In our transcript data (Figure 4C), we show a range of BK transcripts including the Lrrcx subunits, but none, on their own, show significant alteration by treatment. Note that with experimentally elevated intracellular Ca^2+^ concentration these would likely lie well to the left of where they lay in our experiments. In our experiments we included 5 mM EGTA, which allows for the Ca^2+^-activation of BK channels so long as they are physically close to the Ca^2+^ source (Fakler and Adelman, 2008). Functional coupling between BK and the Ca^2+^ source appears a common phenomenon (Fakler and Adelman, 2008); we also showed this with a coupling between TRPV4 channels and *K*_Ca_ channels previously in neurones (Feetham et al., 2015) and it has also been shown in smooth muscle cells too (Nilius and Droogmans, 2001). Increasing, or attempting to “clamp” intracellular Ca^2+^ before investigating BK channels would be tempting, but this could cause greater constitutive activation of BK and mask a physiological coupling.

Our electrophysiological experiments also demonstrated significantly more depolarized resting membrane potentials in cytokine treated cells. This could result from loss of constitutive potassium or chloride conductances, but equally it could result from the elevation of the non-specific or Na^+^ selective or ion channels such as Trpc3 or Asic2, etc. (Table 2).

The BK pharmacological activator (NS1619) and inhibitor (paxilline) both had the expected effects (increase and decrease of current density, respectively) in the untreated FLS but neither had a significant effect. This is surprising in the light of the Tanner et al. (2015) data showing that paxilline ameliorate development joint degeneration in a model of RA and the continued effectiveness of paxilline in FLS from RA patients (Petho et al., 2016). One possible explanation could be differences in transcript expression between strains as has been observed with ion channels previously (Kunert-Keil et al., 2006). Our RNA data showed a clear shift from RNA-expression of β1 (and β2) subunits to β3 and sensitivity to voltage, calcium and some drugs is well-known to be conveyed by co-expression of the β subunits (McManus et al., 1995; Uebele et al., 2000; Yang et al., 2009). However, neither paxilline nor NS1619 themselves are thought to be influenced by the β-subunit; paxilline acts as a closed channel blocker (Zhou and Lingle, 2014) whereas NS1619 opens the BK channel by binding to the KCNMA1 S6/RCK linker and is effective in some splice variants, but not others (Soom et al., 2008; Gessner et al., 2012). In humans there are 93 known s[lice variants of KCNMA1, but there are only four in rats listed on ENSEMBL (Zerbino et al., 2018). Figure 4 shows the relative transcript expression for Kcnma1. The most abundant transcript (ENSRNOT00000077671 Kcnma1-203) is present in both conditions. None of the Kcnma1 transcripts decrease with cytokine so it seems unlikely (but not impossible) that pharmacological changes result directly from splice variant switching. Note that the one known transcript that was not detected is the truncated transcript Kcnma1-204 (ENSRNOT00000091318). The lack of effect of NS1619 and paxilline after cytokine treatment could be also be secondary to changes in β-subunits or intracellular Ca^2+^ in microdomains or some unknown reason leaving insufficient residual functional BK activity to be noticeably modulated. The simple (K^+^ channel focused) FLS electrophysiological model of Clark allows us to use numerical simulation to estimate the change in BK channel Ca^2+^ that would be required to shift a BK conductance voltage curve by about +40 mV and largely leave the cells virtually free of measurable BK current (Figure 6G). We find that retaining all the parameters of Clark et al. (2017) except the Ca^2+^ *Kd* itself, such data would be the equivalent to increasing *Kd* from 0.46 μM to approximately 1.05 μM. The upstream pathway (beyond the activation of the cytokine pathway including NFκB, etc.) for these changes is difficult to identify from our data, especially since there are relatively few ion channel interaction data in the IPA databases. It is notable, however, that there were significant changes in the calcium signaling pathway (Figure 3) and so it is possible that change in calcium activated potassium channel expression follows this, by way of compensatory expression. The lack of apparent effect of paxilline could also be technical. For example, there was considerable variability between cells and we compared population means rather than pairing each cell with and without paxilline increasing the risk of a type II error.

### Role of Ion Channels in Pro-inflammatory Cytokine Production and Secretion

Ion channels are involved not just in the response to cytokines, but can also contribute to their production. For example, ionotropic NMDA and kainite glutamate receptors contribute to synovial inflammation by increasing expression of the inflammatory cytokine IL-6 (Flood et al., 2007) and nicotinic acetylcholine receptor activation reduces the synovial production of IL-6, IL-8, TNFα, and several other cytokines (Waldburger et al., 2008; van Maanen et al., 2009). P2X7 is an established conduit for release of mediators such as IL1β (Mortaz et al., 2012). We saw little P2X7 RNA, but there is evidence that in chondrocytes P2X1, may sub serve the same function (Varani et al., 2008) and we detected RNA for this channel only after cytokine treatment. In a previous report, inhibition of the small Ca^2+^ activated potassium ion channel decreased the production of cytokines IL-6, IL-8, and MCP1 in response to TGF-1β, but they did not examine secretion of IL1β or TNFα (Friebel et al., 2014). In other-words activation of Ca^2+^ potassium ion channels is essentially a secretion trigger. This result is somewhat counter intuitive since one would expect activation of a potassium channel to hyperpolarize and decrease secretion. One possible explanation is that activation of Ca^2+^ potassium ion channels draws in additional Ca^2+^ by increasing the driving force for Ca^2+^ entry.

Caentry2+α(Vm-Eq)Ca2+

Where negative values are equivalent to inward driving force for Ca^2+^, *Vm* is the membrane potential and *Eq_Ca__2__+_* is the equilibrium potential for Ca^2+^.

## Conclusion

To our knowledge, this is the first report using a combined NGS and patch-clamp electrophysiological approach to understanding the control of potassium channels in inflammation in joint tissues. We found an increased RNA expression of the BK potassium gene Kcnma1, but the constitutive activity of the channel was not increased. The decreased sensitivity to voltage activation and to drugs could be explained by a switch the RNA expression of β-subunits KcnmB1, 2, and 3.

## Data Availability Statement

The datasets generated for this study can be found in the https://www.ebi.ac.uk/arrayexpress/experiments/E-MTAB-7798/.

## Ethics Statement

The animal study was reviewed and approved by the University of Liverpool Veterinary Ethics Committee.

## Author Contributions

All authors have conceptualized and designed the study, and analyzed and interpreted the data. RB-J, FO, and KK drafted the manuscript. All authors have critically revised the manuscript for important intellectual content. All authors have finally approved the manuscript. RB-J, AM, and OH obtained the funding.

## Conflict of Interest

The authors declare that the research was conducted in the absence of any commercial or financial relationships that could be construed as a potential conflict of interest.
