# Supplementary material for: Pro-inflammatory Cytokines Drive Deregulation of Potassium Channel Expression in Primary Synovial Fibroblasts
Source: Front Physiol. 2020 Mar 24;11:226. doi: 10.3389/fphys.2020.00226 (PMC7105747; doi:10.3389/fphys.2020.00226)
Supplement: Supplementary file 1 [file Table_1.DOCX]

**Table S1 Genes with the highest expression levels in control treated FLS cells.**

| **Rank** | **Gene symbol**  **(rank in cytokine)** | **Official name*** | **FPKM/1000 (CI)**** |
| --- | --- | --- | --- |
| 1 | Col3a1 (18) | collagen type III alpha 1 chain | 5138 (3314-7965) |
| 2 | Sparc (5) | secreted protein acidic and cysteine rich | 4519 (3355-6085) |
| 3 | Eef2 (1) | eukaryotic translation elongation factor 2 | 3993 (2951-5404) |
| 4 | Serpine1 (2) | serpin family E member 1 | 3721 (2295-6035) |
| 5 | Bgn (3) | biglycan | 3455 (3050-3914) |
| 6 | Actb (4) | β-actin | 2898 (2099-4003) |
| 7 | Thbs1 (48) | thrombospondin 1 | 2388 (1031-5532) |
| 8 | Rplp0 (8) | Large ribosomal protein, P0 | 2668 (2256-3155) |
| 9 | Thbs2 (14) | thrombospondin 2 | 2218 (1483-3316) |
| 10 | Gja1 (24) | gap junction protein α1 | 2265 (1678-3057) |

Ordering from cuffdiff (see methods). *Supplied by the Rat Genome Project <https://rgd.mcw.edu/nomen/nomen.shtml>. **Geometric mean and 95% CI *n=*4.

**Table S2 Genes with the highest expression levels in cytokine (10ng/ml TNFα and IL1β ) treated FLS cells. I**

| **Rank** | **Gene symbol**  **(rank in control)** | **Official name*** | **FPKM/1000 (CI)**** |
| --- | --- | --- | --- |
| 1 | Eef2 (3) | eukaryotic translation elongation factor 2 | 4705 (3644-6076) |
| 2 | Serpine1 (4) | serpin family E member 1 | 2801 (1427-5497) |
| 3 | Bgn (5) | biglycan | 3177 (2635-3831) |
| 4 | Dcn (35) | decorin | 2486 (1100-5617) |
| 5 | Sparc (2) | secreted protein acidic and cysteine rich | 2609 (1509-4511) |
| 6 | Lrp1 (11) | LDL receptor related protein 1 | 2305 (1147-4630) |
| 7 | Ctsb (13) | cathepsin B | 2495 (2027-3071) |
| 8 | Rplp0 (8) | Large ribosomal protein, P0 | 2487 (1906-3245) |
| 9 | Actb (6) | β-actin | 2401 (2028-2844) |
| 10 | Grem1 (16) | gremlin 1, DAN family BMP antagonist | 1985 (967-4076) |

Ordering from cuffdiff (see methods). *Supplied by the Rat Genome Project <https://rgd.mcw.edu/nomen/nomen.shtml>. **Geometric mean and 95% CI *n=*4.

**Table** **S3** **Channel genes with the highest RNA expression levels in control FLS cells**.

| **Rank in Control** | **Gene**  **(rank in cytokine)** | **Official name*** | **FPKM/1000 (CI)**** |
| --- | --- | --- | --- |
| 1 | Gja1 (1) | Gap junction channels | 2265 (1678-3057) |
| 2 | aqp1 (6) | aquaporin 1 (Colton blood group) | 369 (223-609) |
| 3 | piezo1 (2) | piezo type mechanosensitive ion channel component 1 | 289 (236-355) |
| 4 | vdac2 (4) | voltage dependent anion channel 2 | 175 (157-195) |
| 5 | vdac1 (5) | voltage dependent anion channel 1 | 192 (142-258) |
| 6 | clic1 (10) | chloride intracellular channel 1 | 150 (117-191) |
| 7 | trpv2 (9) | transient receptor potential cation channel subfamily v member 2 | 130 (85-199) |
| 8 | p2rx4 (11) | purinergic receptor p2x 4 | 89 (67-117) |
| 9 | clic4 (14) | chloride intracellular channel 4 | 90 (77-105) |
| 10 | trpm7 (12) | transient receptor potential cation channel subfamily m member 7 | 77 (72-82) |
| 11 | clcn7 (15) | chloride voltage-gated channel 7 | 62 (55-70) |
| 12 | cacnb3 (13) | calcium voltage-gated channel auxiliary subunit beta 3 | 44 (28-71) |
| 13 | kcnk2 (21) | potassium two pore domain channel subfamily k member 2 | 47 (37-58) |
| 14 | tpcn1 (17) | two pore segment channel 1 | 49 (40-61) |
| 15 | clcc1 (16) | chloride channel clic like 1 | 44 (40-49) |
| 16 | scn1b (22) | sodium voltage-gated channel beta subunit 1 | 41 (31-55) |
| 17 | clns1a (19) | chloride nucleotide-sensitive channel 1a | 40 (29-54) |
| 18 | clcn6 (20) | chloride voltage-gated channel 6 | 32 (25-40) |
| 19 | kcnq5 (18) | potassium voltage-gated channel subfamily q member 5 | 27 (18-39) |
| 20 | cacfd1 (24) | calcium channel flower domain containing 1 | 24 (21-28) |
| 21 | trpm4 (25) | transient receptor potential cation channel subfamily m member 4 | 20 (17-24) |
| 22 | clcn5 (26) | chloride voltage-gated channel 5 | 20 (17-23) |
| 23 | Gjb2 (42) | Gap junction channels | 16 (10-26) |
| 24 | kcnd1 (32) | potassium voltage-gated channel subfamily d member 1 | 14 (12-17) |
| 25 | kcnk12 (35) | potassium two pore domain channel subfamily k member 12 | 10 (3-29) |
| 26 | clcn3 (27) | chloride voltage-gated channel 3 | 10 (7-14) |
| 27 | Gjc1 (33) | Gap junction channels | 10 (7-13) |
| 28 | kcnd3 (38) | potassium voltage-gated channel subfamily d member 3 | 7 (3-16) |
| 29 | clic2 (45) | chloride intracellular channel 2 | 8 (6-12) |
| 30 | cacna1g (43) | calcium voltage-gated channel subunit alpha1 g | 8 (6-11) |
| 31 | cacna1a (34) | calcium voltage-gated channel subunit alpha1 a | 7 (5-10) |
| 32 | cacna1c (36) | calcium voltage-gated channel subunit alpha1 c | 7 (4-11) |
| 33 | scnm1 (40) | sodium channel modifier 1 | 7 (6-8) |
| 34 | hvcn1 (23) | hydrogen voltage gated channel 1 | 7 (6-8) |
| 35 | p2rx5 (37) | purinergic receptor p2x 5 | 5 (2-13) |
| 36 | kcna4 (31) | potassium voltage-gated channel subfamily a member 4 | 4 (1-14) |
| 37 | tpcn2 (39) | two pore segment channel 2 | 6 (5-8) |
| 38 | Gja5 (28) | Gap junction channels | 2 (0-11) |
| 39 | cacna1b (44) | calcium voltage-gated channel subunit alpha1 b | 4 (3-6) |
| 40 | trpm3 (47) | transient receptor potential cation channel subfamily m member 3 | 3 (1-6) |
| 41 | trpm1 (53) | transient receptor potential cation channel subfamily m member 1 | 2 (1-7) |
| 42 | kcnn4 (52) | potassium calcium-activated channel subfamily n member 4 | 3 (2-5) |
| 43 | kcnma1 (41) | potassium calcium-activated channel subfamily m alpha 1 | 2 (1-6) |
| 44 | trpv4 (50) | transient receptor potential cation channel subfamily v member 4 | 3 (2-4) |
| 45 | kcne4 (49) | potassium voltage-gated channel subfamily e regulatory subunit 4 | 2 (1-6) |
| 46 | catsper2 (55) | cation channel sperm associated 2 | 3 (2-3) |
| 47 | scn2a (58) | sodium voltage-gated channel alpha subunit 2 | 2 (2-3) |
| 48 | kcnj2 (60) | potassium inward rectifier j member 2 | 2 (1-3) |
| 49 | clic3 (57) | chloride intracellular channel 3 | 2 (2-2) |
| 50 | ano1 (75) | anoctamin 1 | 0 (0-1) |

Ordering from cuffdiff (see methods). *Supplied by the Rat Genome Project <https://rgd.mcw.edu/nomen/nomen.shtml>. **Geometric mean and 95% CI *n=*4.

**Table S4 Channel genes with the highest RNA expression levels in cytokine (10ng/ml TNFα and IL1β ) treated FLS cells.**

| **Rank** | **Gene**  **(control rank)** | **Official name*** | **FPKM/1000 (CI)**** |
| --- | --- | --- | --- |
| 1 | Gja1 (1) | Gap junction channels | 1489 (930-2385) |
| 2 | piezo1 (3) | piezo type mechanosensitive ion channel component 1 | 276 (247-308) |
| 3 | vdac2 (5) | voltage dependent anion channel 2 | 181 (142-233) |
| 4 | vdac1 (6) | voltage dependent anion channel 1 | 227 (169-306) |
| 5 | aqp1 (2) | aquaporin 1 (colton blood group) | 144 (86-242) |
| 6 | vdac3 (7) | voltage dependent anion channel 1 pseudogene 5 | 155 (121-200) |
| 7 | trpv2 (9) | transient receptor potential cation channel subfamily v member 2 | 115 (77-172) |
| 8 | clic1 (8) | chloride intracellular channel 1 | 125 (108-146) |
| 9 | p2rx4 (10) | purinergic receptor p2x 4 | 85 (52-140) |
| 10 | trpm7 (13) | transient receptor potential cation channel subfamily m member 7 | 85 (75-97) |
| 11 | clic4 (11) | chloride intracellular channel 4 | 79 (68-93) |
| 12 | clcn7 (14) | chloride voltage-gated channel 7 | 64 (52-79) |
| 13 | clcc1 (18) | chloride channel clic like 1 | 52 (42-65) |
| 14 | tpcn1 (17) | two pore segment channel 1 | 56 (48-66) |
| 15 | kcnq5 (22) | potassium voltage-gated channel subfamily q member 5 | 34 (17-68) |
| 16 | clns1a (20) | chloride nucleotide-sensitive channel 1a | 36 (34-38) |
| 17 | clcn6 (21) | chloride voltage-gated channel 6 | 34 (27-44) |
| 18 | kcnk2 (16) | potassium two pore domain channel subfamily k member 2 | 25 (12-49) |
| 19 | scn1b (19) | sodium voltage-gated channel beta subunit 1 | 28 (19-42) |
| 20 | hvcn1 (39) | hydrogen voltage gated channel 1 | 22 (9-55) |
| 21 | cacfd1 (23) | calcium channel flower domain containing 1 | 27 (24-30) |
| 22 | trpm4 (24) | transient receptor potential cation channel subfamily m member 4 | 19 (13-27) |
| 23 | clcn5 (25) | chloride voltage-gated channel 5 | 19 (16-23) |
| 24 | clcn3 (31) | chloride voltage-gated channel 3 | 15 (9-27) |
| 25 | Gja5 (43) | Gap junction channels | 2 (0-20) |
| 26 | kcna4 (41) | potassium voltage-gated channel subfamily a member 4 | 4 (0-32) |
| 27 | kcnd1 (27) | potassium voltage-gated channel subfamily d member 1 | 12 (9-15) |
| 28 | Gjc1 (32) | Gap junction channels | 11 (9-13) |
| 29 | cacna1a (36) | calcium voltage-gated channel subunit alpha1 a | 11 (9-13) |
| 30 | kcnk12 (28) | potassium two pore domain channel subfamily k member 12 | 8 (3-20) |
| 31 | cacna1c (37) | calcium voltage-gated channel subunit alpha1 c | 7 (3-19) |
| 32 | p2rx5 (40) | purinergic receptor p2x 5 | 6 (2-18) |
| 33 | kcnd3 (33) | potassium voltage-gated channel subfamily d member 3 | 2 (0-12) |
| 34 | tpcn2 (42) | two pore segment channel 2 | 7 (5-10) |
| 35 | scnm1 (38) | sodium channel modifier 1 | 6 (6-8) |
| 36 | kcnma1 (49) | potassium calcium-activated channel subfamily m alpha 1 | 2 (0-13) |
| 37 | Gjb2 (26) | Gap junction channels | 4 (1-12) |
| 38 | cacna1g (35) | calcium voltage-gated channel subunit alpha1 g | 5 (4-6) |
| 39 | cacna1b (44) | calcium voltage-gated channel subunit alpha1 b | 5 (3-6) |
| 40 | clic2 (34) | chloride intracellular channel 2 | 3 (1-8) |
| 41 | trpm3 (46) | transient receptor potential cation channel subfamily m member 3 | 3 (1-6) |
| 42 | kcne4 (51) | potassium voltage-gated channel subfamily e regulatory subunit 4 | 2 (1-7) |
| 43 | trpv4 (50) | transient receptor potential cation channel subfamily v member 4 | 3 (1-6) |
| 44 | kcnn3 (72) | potassium calcium-activated channel subfamily n member 3 | 1 (0-7) |
| 45 | kcnn4 (48) | potassium calcium-activated channel subfamily n member 4 | 2 (1-6) |
| 46 | trpm1 (47) | transient receptor potential cation channel subfamily m member 1 | 2 (1-5) |
| 47 | noct (57) | anoctamin 1 | 3 (2-3) |
| 48 | catsper2 (52) | cation channel sperm associated 2 | 2 (2-3) |
| 49 | kcnn1 (61) | potassium calcium-activated channel subfamily n member 1 | 2 (2-2) |
| 50 | clic3 (56) | chloride intracellular channel 3 | 2 (1-2) |

Ordering from cuffdiff (see methods). *Supplied by the Rat Genome Project <https://rgd.mcw.edu/nomen/nomen.shtml>. **Geometric mean and 95% CI *n=*4.

**Table S5 All channel genes “appearing” in cytokine (10ng/ml TNFα and IL1β ) treated FLS cells (undetectable in control).**

| **Gene** | **Official Name** | **FPKM (95% CI)** |
| --- | --- | --- |
| kcnip2 | potassium voltage-gated channel interacting protein 2 | 166.5 (110.9-249.9) |
| trpc3 | transient receptor potential cation channel subfamily c member 3 | 8.3 (0.7-98.2) |
| kcng3 | potassium voltage-gated channel modifier subfamily g member 3 | 7 (0.7-71.7) |
| scnn1g | sodium channel epithelial 1 gamma subunit | 5.9 (0.7-46.8) |
| kcnmb3 | potassium calcium-activated channel subfamily m regulatory beta subunit 3 | 5.1 (0.8-33.1) |
| Gja3 | Gap junction type a3 | 4.6 (0.8-27.3) |
| kcnj11 | potassium inward rectifier j member 11 (Kir6.2) | 3.3 (0.3-37.4) |
| asic5 | acid sensing ion channel subunit family member 5 | 2.9 (0.3-24.9) |
| clca1 | chloride channel accessory 1 | 2.6 (0.4-18.4) |
| kcnc2 | potassium voltage-gated channel subfamily c member 2 | 2.6 (0.4-18.4) |
| kcnj5 | potassium voltage-gated channel subfamily j member 5 | 2.6 (0.4-18.4) |
| trpm2 | transient receptor potential cation channel subfamily m member 2 | 2.6 (0.4-18.4) |
| cacna2d4 | calcium voltage-gated channel auxiliary subunit alpha2delta 4 | 2.5 (0.4-15.3) |
| kcnk13 | potassium two pore domain channel subfamily k member 13 | 2.5 (0.4-15.3) |
| trpc4 | transient receptor potential cation channel subfamily c member 4 | 2.2 (0.4-11.2) |
| p2rx1 | purinergic receptor p2x 1 | 2.2 (0.5-10.9) |
| scn11a | sodium voltage-gated channel alpha subunit 11 | 2.2 (0.5-10.9) |
| Gja8 | Gap junction type a8 | 2.1 (0.5-9.1) |
| kcnj13 | potassium voltage-gated channel subfamily j member 13 | 1 (1-1) |

Data are presented in rank order; top FPKM genes in the presence of cytokine first. None had any expression in control (i.e., non-cytokine treated = 0±0 FPKM *n*=4). Overall, n=4 treated and 4 control animals.

**Table 6 Channel genes “disappearing” in cytokine (10ng/ml TNFα and IL1β ) treated FLS cells (undetectable in cytokine).**

| **Gene** | **Official Name** | **FPKM (95% CI)** |
| --- | --- | --- |
| ano2 | anoctamin 2 | 6 (0.8-48.5) |
| kcnv2 | potassium voltage-gated channel subfamily a member 2 | 5.3 (0.8-36.3) |
| kcna2 | potassium voltage-gated channel modifier subfamily v member 2 | 2.8 (0.4-21) |
| kcnmb2 | potassium calcium-activated channel subfamily m regulatory beta subunit 2 | 2.6 (0.4-18.2) |
| trpm5 | transient receptor potential cation channel subfamily m member 5 | 2.3 (0.4-12.5) |
| kcne5 | potassium voltage-gated channel subfamily e regulatory subunit 5 | 2.3 (0.4-11.9) |
| cacna1e | calcium voltage-gated channel subunit alpha1 e | 2.2 (0.5-10.4) |

Data are presented in rank order; top FPKM genes in the presence of cytokine first. None had any expression in cytokine treated animals (i.e., cytokine treated = 0±0 FPKM *n*=4). Overall, n=4 treated and 4 control animals.
