# Supplementary material for: Pro-inflammatory Cytokines Drive Deregulation of Potassium Channel Expression in Primary Synovial Fibroblasts
Source: Front Physiol. 2020 Mar 24;11:226. doi: 10.3389/fphys.2020.00226 (PMC7105747; doi:10.3389/fphys.2020.00226)
Supplement: Supplementary file 2 [file Data_Sheet_1.docx]

%% Simulation included in Haidar et al 2019, written by RBJ

%% Based on Clark et al 2017 equations

global T F R k e Ki Ko Cai GKir

global aKir bKir GBK L0 zL J0 zJ kd C D Gb Eb E EK

T=294;

F=96500;

R=8.31;

k=1.38E-23;

e=1.6E-19;

Ki=120;

Ko=5;

Cai=129.92e-9; %Clark ->Giles say 129.92e-9

%Cai=5e-9; % assuming 0.1% Ca contamination in the intracellular salt

GKir=23.15;

aKir=0.94;

bKir=1.26;

GBK=1784.19;

L0=0.00000000263;

zL=0.95;

J0=4237.36;

zJ=1.05;

C=8;

D=25;

Gb=10.56;

Eb=-21.51;

E=2.5; %the Aldrich paper mentions this

EK=-85;

kd=1.1e-7; %Wayne (Clarke Rob) said 11e-6

hold off

Vm=(-50.0:0.1:180.0);

localkd=kd;

inc=1;

maxi=1000;

lenny=maxi/inc;

results=zeros(lenny,5);

j=0;

for i=1:inc:maxi

j=j+1;

kd=localkd*double(i)/10.0;

results(j,1)=kd*1e6;%micromolar

IFIB=getIBK(Vm)+getIb(Vm) +getIKR(Vm);

cond=1000.*IFIB./(Vm-EK);

subplot(2,1,1);

hold on

plot(Vm,IFIB);

subplot(2,1,2);

hold on

cond=cond./max(cond);

%midpoint

dum=cond-0.5;

[c,index]=min(abs(dum));

Midpoint=Vm(index);

results(j,2)=Midpoint;

plot(Vm,cond);

[c, index] = min(abs(IFIB));

kd

vrev=Vm(index)

results(j,3)=vrev;

end

subplot(2,1,1);

line(xlim, [0,0], 'LineWidth', 2, 'Color', 'k');

line([0,0], ylim, 'LineWidth', 2, 'Color', 'k');

subplot(2,1,2);

line(xlim, [0,0], 'LineWidth', 2, 'Color', 'k');

line([0,0], ylim, 'LineWidth', 2, 'Color', 'k');

disp('end');

function rtn=getPo(Vm)

global Cai kd J0 zJ k T zL C D E e L0;

K=Cai/kd;

exper=e*Vm./(k*T*1000);

J=J0*exp(-zJ.*exper);

L=L0*exp(zL.*exper);

top=L.*(1+K*C+J.*D+J.*K*C*D*E).^4;

bot_left=L.*(1+K*C+J.*D+J.*K*C*D*E).^4;

bot_right=(1+J+K+J*K*E).^4;

rtn=top./(bot_left+bot_right);

end

function rtn=getIBK(Vm)

global GBK EK ;

rtn=GBK.*getPo(Vm).*(Vm-EK)./1000.0;

end

function rtn=getIb(Vm)

global Gb Eb;

rtn=Gb.*(Vm-Eb)./1000.0;

end

function rtn=getIKR(Vm)

global Ko aKir bKir EK F R T GKir

vdif=(Vm-EK)./1000.0;

expr=exp(bKir.*vdif.*F./(R*T));

bot=aKir+expr;

Kos=Ko^0.5;

rtn=GKir*Kos*(1./bot).*(Vm-EK)./1000.0;

end
