# Supplementary material for: Pro-inflammatory Cytokines Drive Deregulation of Potassium Channel Expression in Primary Synovial Fibroblasts
Source: Front Physiol. 2020 Mar 24;11:226. doi: 10.3389/fphys.2020.00226 (PMC7105747; doi:10.3389/fphys.2020.00226)
Supplement: Supplementary file 3 [file Data_Sheet_2.docx]

**Supplementary Methods Details**

es were then incubated for 1 h at room temperature in tris-

buffered saline (TBS) containing 1% bovine serum albumin (BSA,

protease-free) and 0.01% sodium azide to block non-speciﬁc

antibody binding.

The

slides were then incubated for 1 h at room temperature in tris-

buffered saline (TBS) containing 1% bovine serum albumin (BSA,

protease-free) and 0.01% sodium azide to block non-speciﬁc

antibody binding.

The

slides were then incubated for 1 h at room temperature in tris-

buffered saline (TBS) containing 1% bovine serum albumin (BSA,

protease-free) and 0.01% sodium azide to block non-speciﬁc

antibody binding.

**NGS Additional Details**

For quality control, we calculated phred quality scores with the RSeQC package (Wang *et al.* 2012) using a cut off of 37. Reads were aligned and mapped with the Hisat2 program (Kim *et al.* 2015) gene expression was quantified using the StringTie transcript assembly and quantification package (Johns Hopkins University, USA). This package uses the resulting BAM and annotation (GFF3) files as input to generate; assembled transcripts, gene abundance estimates [normalized FPKM (Fragments per kilobase exon per million reads mapped) and normalized TPM (Transcripts per kilobase million)] and coverage data. Any transcripts with <200 nucleotides in length were removed. Values are displayed as geometric means (95% confidence intervals).

Global gene differential expression was determined with CuffDiff (Broad Institute of MIT and Harvard, USA), assessing differences between groups, yielding log2 fold change, p-values and adjusted q-values (False Discovery Rate (FDR)). For library normalization, geometric method was used. The FDR was less than 0.05. Ingenuity Pathway Analysis (IPA, Qiagen, Manchester, UK) was used to for network analysis and for calculation of causal regulators.

Ion channel genes were detected in the global gene expression data using custom MATLAB scripts cross referencing gene IDs to HUGO Gene descriptors (using Genenames.org, HUGO Gene Nomenclature Committee). For differential expression ratios, a minimum level threshold of unity was applied (Kuehn *et al.* 2008). Differential expression ratios confidence intervals were calculated with the bootstrap method (implemented in MatLab, 10,000 samples per gene).

Libraries of RNA sequenced samples were generated and analysis took place using a bioinformatics platform <https://usegalaxy.org> website.

***Drugs***

All reagents were purchases from Sigma-Aldrich, UK, unless otherwise stated.

***Statistics***

Data are expressed as mean ± SD, where n=3, or mean ± SEM, where n ≥ 4. Differences between mean values were assessed using Student’s t-test. Where multiple treatments were compared, ANOVA followed by a modified t-test was used. Curve fitting and calculation of F-tests were performed with Matlab. P < 0.05 was considered statistically significant.

**References**

Kim, D., Langmead, B. and Salzberg, S.L. (2015) 'HISAT: a fast spliced aligner with low memory requirements', *Nat Methods*, 12(4), 357-60, available: <http://dx.doi.org/10.1038/nmeth.3317>.

Kuehn, H., Liberzon, A., Reich, M. and Mesirov, J.P. (2008) 'Using GenePattern for gene expression analysis', *Curr Protoc Bioinformatics*, Chapter 7, Unit 7 12, available: <http://dx.doi.org/10.1002/0471250953.bi0712s22>.

Wang, L., Wang, S. and Li, W. (2012) 'RSeQC: quality control of RNA-seq experiments', *Bioinformatics*, 28(16), 2184-5, available: <http://dx.doi.org/10.1093/bioinformatics/bts356>.
